# Evaluation of antibacterial efficiency of *Trapa natans* modified glass ionomer cement: An in vitro study

**DOI:** 10.1016/j.jtumed.2026.04.005

**Published:** 2026-05-07

**Authors:** Ayesha Imtiaz, Shaukat Khalid, Zubair Anwar, Rida Fatima Khan, Muhammad Khawaja Hammad Uddin, Affan Ahmad

**Affiliations:** a Department of Dental Material, Baqai Medical University, Pakistan; b Department of Pharmacognosy, Baqai Medical University, Pakistan; c Department of Pharmaceutical Chemistry, Baqai Medical University, Pakistan; d Department of Oral Pathology, Dow Dental College, Dow University of Health Science, Pakistan; e Department of Dental Materials, Dow University of Health Science, Pakistan; f Department of Science of Dental Materials, Karachi Medical and Dental College, Karachi Metropolitan University, Pakistan

**Keywords:** إسمنت الزجاج الأيوني, الكستناء المائية, النشاط المضاد للبكتيريا, المكورات العقدية الطافرة, الإشريكية القولونية, Antibacterial activity, *Escherichia coli*, Glass ionomer cement, *Streptococcus mutans*, *Trapa natans*

## Abstract

**Objective:**

To evaluate the antibacterial efficacy of incorporating Trapa natans (water chestnut) powder at 0%, 1%, 2%, and 5% (w/w) into glass ionomer cements (GICs), GC Gold Label 2 and Shofu GIC.

**Methods:**

An in vitro agar disc diffusion assay was conducted to assess antibacterial activity against Streptococcus mutans, Lactobacillus spp., and Escherichia coli. Zones of inhibition (mm ± standard deviation) were measured after 24 and 48 hours. Data were analyzed using two-way analysis of variance followed by post hoc Tukey tests.

**Results:**

The highest antibacterial activity for GC Gold Label 2 at 24 hours was observed at 2% concentration (2.20 ± 0.10 mm), followed by 5% (2.16 ± 0.10 mm), control (2.13 ± 0.10 mm), and 1% (2.10 ± 0.10 mm). Shofu GIC showed greater activity at 2% (2.43 ± 0.10 mm), followed by 5% (2.32 ± 0.10 mm), 1% (2.30 ± 0.10 mm), and control (2.20 ± 0.10 mm). Antibacterial effects increased further at 48 hours. Statistically significant differences were observed for 2% and 5% concentrations compared with control in both GIC brands (p < 0.05).

**Conclusions:**

Incorporation of Trapa natans powder significantly improved the antibacterial properties of conventional GICs in a concentration- and time-dependent manner, indicating its potential as a natural additive to enhance the bioactivity of restorative dental materials.

## الملخص

**أهداف البحث:**

هدفت الدراسة إلى تقييم الفعالية المضادة للبكتيريا لإضافة مسحوق نبات الكستناء المائية إلى إسمنتي الزجاج الأيوني GC Gold Label 2 وShofu GIC بتراكيز 0% و1% و2% و5% من الوزن الكلي.

**طرق البحث:**

أُجريت اختبارات مخبرية باستخدام تقنية انتشار الأقراص على الأوساط الزرعية الصلبة لتقييم النشاط المضاد للبكتيريا ضد المكورات العقدية الطافرة، وأنواع العصيات اللبنية، والإشريكية القولونية. وقيسَت أقطار مناطق التثبيط بالملليمتر مع الانحراف المعياري بعد 24 ساعة و48 ساعة. وقورنت النتائج باستخدام تحليل التباين الثنائي، تلاه اختبار توكي للمقارنات البعدية.

**النتائج:**

سُجِّلت أكبر منطقة تثبيط مجمعة بعد 24 ساعة في مجموعة GC Gold Label 2 عند تركيز 2%، حيث بلغت 2.20 ± 0.10 مم، تلاها تركيز 5% بمقدار 2.16 ± 0.10 مم، ثم المجموعة الضابطة بتركيز 0% بمقدار 2.13 ± 0.10 مم، ثم تركيز 1% بمقدار 2.10 ± 0.10 مم. أما Shofu GIC فقد أظهر فعالية أعلى عند تركيز 2%، حيث بلغت 2.43 ± 0.10 مم، تلاه تركيز 5% بمقدار 2.32 ± 0.10 مم، ثم تركيز 1% بمقدار 2.30 ± 0.10 مم، ثم المجموعة الضابطة بتركيز 0% بمقدار 2.20 ± 0.10 مم. وقد تعزّز التأثير المضاد للبكتيريا في جميع المعالجات التجريبية بعد 48 ساعة. كما ظهرت فروق ذات دلالة إحصائية بين تركيزي 2% و5% في كلا النوعين من إسمنت الزجاج الأيوني مقارنةً بالمجموعة الضابطة.

**الاستنتاجات:**

أظهرت إضافة مسحوق الكستناء المائية تحسنًا ملحوظًا في النشاط المضاد للبكتيريا لإسمنتات الزجاج الأيوني التقليدية بطريقة تعتمد على التركيز والزمن، مما يدعم إمكانية استخدامه كمضاف طبيعي لتحسين الفعالية الحيوية لمواد ترميم الأسنان.

## Introduction

Dental caries remains the most prevalent non-communicable disease worldwide, affecting over 3.5 billion individuals.^1^ This multifactorial biofilm-mediated disease is caused primarily by acidogenic and aciduric microorganisms, particularly *Streptococcus mutans*, which metabolize dietary carbohydrates into organic acids, leading to the demineralization of enamel.^2^ In addition to *S. mutans* and *Lactobacillus* spp., other opportunistic pathogens such as *Staphylococcus aureus*, *Pseudomonas aeruginosa*, *Escherichia coli*, and *Candida albicans* have been implicated in the progression of dental caries and secondary infections.^3^, ^4^, ^5^

In Pakistan, approximately 60% of the population is affected by dental caries,^6^ contributing to a substantial socioeconomic burden. Globally, oral diseases accounted for an estimated US$710 billion in 2019 due to treatment costs and productivity losses.^7^ Consequently, minimal intervention strategies such as atraumatic restorative treatment have gained attention. These approaches preferentially utilize glass ionomer cements (GICs) due to their chemical adhesion to the tooth structure, fluoride release, biocompatibility, and ease of handling.^3^

Conventional GICs initially exhibit antibacterial activity due to fluoride release and an acidic pH but this effect diminishes over time. Consequently, the long-term antibacterial performance of conventional GICs may be insufficient to prevent secondary caries. Secondary caries remains a major cause of restoration failure, and thus there is a need for restorative materials with sustained antimicrobial properties. Therefore, it is clinically relevant to explore natural bioactive modifiers that can enhance the long-term antibacterial performance of GICs without compromising the integrity of these materials.^8^

Recent research has focused on enhancing the antibacterial activity of GICs by incorporating natural extracts with known antimicrobial properties.^9^ In particular, *Trapa natans* (water chestnut), an aquatic medicinal plant, has antioxidant, anti-inflammatory, and antimicrobial activities. Extracts derived from the fruit of *Trapa natans* have exhibited antibacterial efficacy against Gram-negative organisms such as *S. aureus*, *E. coli*, and *Shigella sonnei*.^10^

Phytochemical analyses have demonstrated that *Trapa natans* contains phenolic compounds, flavonoids, tannins, coumarins, and antioxidants, which exert antimicrobial effects by disrupting bacterial cell membranes, denaturing proteins, and interfering with nucleic acid synthesis.^11^ Moreover, the fruit is rich in minerals such as calcium, potassium, and magnesium, which contribute to its bioactive properties.^12^^,^^13^

In addition to its phytochemical constituents, *Trapa natans* has distinctive physicochemical characteristics. The starch extracted from its fruit is approximately 85% nitrogen-free, with minimal protein (0.35%) and ash contents. The amylose and amylopectin fractions were reported to be approximately 23.89% and 76.11%, respectively, indicating a predominance of amylopectin. Fourier-transform infrared spectroscopy confirmed its polysaccharide structure, and X-ray diffraction indicated an orthorhombic crystalline pattern. Scanning electron microscopy detected rough, cuboidal granules, and rheological analysis determined strong gel-forming behavior. These structural and functional properties suggest that *Trapa natans* powder may be compatible with restorative material matrices and capable of uniform dispersion within GICs.

*Trapa natans* contains starch but concerns regarding its cariogenic potential are minimal in this application. After its incorporation into GIC powder at controlled concentrations (1–5% w/w), the starch becomes encapsulated within the hardened cement matrix, limiting its availability as a fermentable substrate for utilization by oral bacteria. Thus, unlike free dietary carbohydrates, the embedded starch is not readily metabolized by cariogenic microorganisms. Moreover, the antimicrobial activities of the phenolic and flavonoid constituents are expected to take precedence over any theoretical cariogenic risk.^14^

In previous studies, *Trapa natans* has not been investigated as a modifier of restorative dental materials, and thus there is insufficient evidence regarding whether its incorporation into GICs can enhance their antibacterial performance. Therefore, in the present study, the antibacterial efficacy was evaluated for *Trapa natans*-modified conventional GICs (GC Gold Label 2 and Shofu) at concentrations of 1%, 2%, and 5% (w/w).

The findings obtained in the present study provide evidence that incorporating *Trapa natans* into conventional GICs may enhance their antibacterial activity against cariogenic pathogens. Thus, clinicians could employ this natural, cost-effective strategy to potentially improve the antimicrobial performance of restorative materials and reduce the risk of secondary caries.

In this study, the null hypothesis (H_0_) was that incorporating *Trapa natans* powder into conventional GICs would not significantly alter their antibacterial activity compared with unmodified GICs.

## Methodology

### Study design and setting

This in vitro experimental study was conducted at Baqai Dental College over a period of 1 year, following approval from the Institutional Ethics Review Committee **(Approval No. BDC/ERC/2023/052 dated 15-June-2023)**.

### Materials

The test material used in this study was dry *Trapa natans* (water chestnut) fruit powder, which was obtained locally from a market, and taxonomic identification (Voucher number: FMRC/Herb/0179/23) was performed at the Pakistan Council of Scientific and Industrial Research, Karachi. In addition, two commercially available GIC brands were used: GC Gold label 2 (GC Corporation, Tokyo, Japan) and Shofu GIC (Shofu Inc., Kyoto, Japan).

### Plant collection procedure

The dried *Trapa natans* (water chestnut) fruit was obtained from a local market and ground into powder using a home blender. This in vitro study ensured that no humans were exposed to the active powdered material. It has been shown that the acute oral toxicity of hydroalcoholic, ethanolic, and methanolic extracts of *Trapa natans* is safe up to a dosage of 2000 mg/kg.^11^

### Preparation of Trapa natans Powder

Fresh Trapa natans (water chestnut) fruits were collected from a local market, thoroughly washed under running tap water, and shade-dried at room temperature for 7 days to preserve phytochemical integrity. The fruits were then oven-dried at 40–45°C until a constant weight was achieved. The dried fruits were ground into fine powder using a mechanical grinder and passed through a 75 μm sieve to obtain a uniform powder suitable for blending with restorative GIC powders. Since this was an in vitro study, no human subjects were exposed to the powdered material. Previous studies have reported that the acute oral toxicity of hydroalcoholic, ethanolic, and methanolic extracts of Trapa natans is safe up to a dosage of 2000 mg/kg.^11^

The dry *Trapa natans* powder was incorporated into two conventional GIC powders (GC Gold 2 and Shofu) at weight percentages of 1%, 2%, and 5% (w/w). Each mixture was homogenized using a mortar and pestle for 10 min to ensure that the plant powder was evenly distributed within the cement matrix. All specimens were prepared according to the manufacturer's recommended powder:liquid ratio of 1:1 for glass ionomer restorative cements.

### Experimental treatments

The experimental design tested eight treatments by incorporating different concentrations of *Trapa natans* (water chestnut) powder into commercially available GICs: GC Gold label 2 (GC Corporation, Tokyo, Japan) and Shofu GIC (Shofu Inc., Kyoto, Japan). Treatment 1A consisted of GC Gold Label 2 without *Trapa natans* and served as the control. Treatments 1B, 1C, and 1D consisted of Gold Label 2 GIC incorporating *Trapa natans* at concentrations of 1%, 2%, and 5% (w/w), respectively. Similarly, treatment 2A consisted of Shofu GIC without *Trapa natans* and served as the control. Treatments 2B, 2C, and 2D consisted of Shofu GIC incorporating *Trapa natans* at concentrations of 1%, 2%, and 5% (w/w), respectively, as shown in Table 1.

**Table 1:** Formulations of experimental treatments using GC Gold Label 2 and Shofu GIC modified with different concentrations of *Trapa natans* fruit powder.

Experimental treatment	GIC brand	*Trapa natans* powder concentration (by weight)	Description
1A	GC Gold Label 2	0% (None)	Control for GC Gold Label 2
1B	GC Gold Label 2	1%	GC Gold Label 2 incorporating 1% *Trapa natans*
1C	GC Gold Label 2	2%	GC Gold Label 2 incorporating 2% *Trapa natans*
1D	GC Gold Label 2	5%	GC Gold Label 2 incorporating 5% *Trapa natans*
2A	Shofu GIC	0% (None)	Control for Shofu GIC
2B	Shofu GIC	1%	Shofu GIC incorporating 1% *Trapa natans*
2C	Shofu GIC	2%	Shofu GIC incorporating 2% *Trapa natans*
2D	Shofu GIC	5%	Shofu GIC incorporating 5% *Trapa natans*

Each formulation was thoroughly homogenized before use in preparing disks for agar disk diffusion assays. The powder and liquid were combined at a ratio of 1:1, according to the manufacturer's instructions. The GIC powder was mixed homogenously with the water chestnut in a mortar and pestle for 10 min. The powder components were weighed precisely using an electronic weighing balance (Kern ALS 220-4, Salford Scientific, UK).

### Sample size determination

Sample sizes were calculated a priori using G∗Power software (version 3.1.9.4, Heinrich-Heine-Universität Düsseldorf, Germany). Two-way fixed-effects analysis of variance (ANOVA) was conducted to evaluate the effects of two independent variables: GIC brand (GC Gold Label 2 or Shofu) and *Trapa natans* concentration (0%, 1%, 2%, or 5%).

Using a large effect size (f = 0.40), significance level (α = 0.05), and power (1− β = 0.80), 72 experimental units were required (numerator df = 3; denominator df = 64; critical F = 2.745), which corresponded to n = 3 experimental units per treatment for each bacterial strain (*S. mutans*, *L. bacillus*, and *E. coli*). Triplicate testing of each disk was performed to ensure reproducibility.

### Preparation of sterile disks

Disks (6 mm diameter) were punched from each GIC formulation and sterilized at 121 °C on Whatman No. 3 filter papers. Disks were air dried at 37 °C for 4 h and stored at −20 °C until use. Nine disks were used per experimental treatment, which corresponded to three disks per bacterial strain.

### Assessment of antibacterial activity

The antimicrobial activities of conventional GICs (GC Gold Label 2 and Shofu) and *Trapa natans*-modified GICs were evaluated against *S. mutans*, *Lactobacillus* spp., and *E. coli* using the agar disk diffusion method. All bacterial strains were standard laboratory strains obtained from the American Type Culture Collection (ATCC, USA).

#### Preparation of culture medium

Mueller Hinton agar (MHA) was prepared by dissolving 17.0 g agar, 1.5 g starch, 17.5 g casein hydrolysate, and 2.0 g beef extract in distilled water. The agar was sterilized in an autoclave at 121 °C for 15 min, before pouring into sterile Petri dishes and allowing to dry under aseptic conditions.

#### Preparation of bacterial inoculum

Bacterial strains were rehydrated, inoculated into nutrient broth, and incubated until visible turbidity indicated active growth. Turbidity was standardized against the 0.5 McFarland standard (∼1.5 × 10^8^colony-forming units/mL) using a mixture obtained by combining 0.05 mL of 1.175% barium chloride dihydrate (BaCl_2_·2H_2_O) with 9.95 mL of 1% sulfuric acid (H_2_SO_4_). Slant cultures were incubated at 24–48 h and actively growing cultures were transferred to nutrient broth to obtain the inoculum.

#### Agar disc diffusion procedure

Each bacterial inoculum was evenly spread on MHA plates using a sterile cotton swab. Sterile forceps were used to place GIC discs on the surface of the agar at equidistant intervals to prevent zones of inhibition from overlapping. Plates were incubated at 37 °C and the zones of inhibition were measured in millimeters after 24 h and 48 h. Each experiment was performed in triplicate to ensure reproducibility. The diameter of the clear zone around each disk indicated the antibacterial activity of the corresponding GIC formulation.

### Statistical analysis

SPSS version 21 was used to analyze the data. Data normality was first checked using the Shapiro–Wilk test, where a *p*-value greater than 0.05 indicated that the data followed a normal distribution. Two-way ANOVA was conducted to assess the effects of the two independent variables, i.e., GIC brand (GC Gold Label 2 and Shofu) and *Trapa natans* concentration (0% control, 1%, 2%, or 5%), on the antibacterial activities based on the zone of inhibition after 24 and 48 h. After ANOVA, post hoc test Tukey tests were performed to detect significant difference in observations between experimental treatments.

## Results

### Antibacterial activities

The antibacterial efficacy was evaluated for *Trapa natans* modified GICs (GC Gold Label 2 and Shofu GIC) against *S. mutans*, *Lactobacillus* spp., and *E. coli* by using the agar disk diffusion technique. The zone of inhibition (mm) was measured after 24 h and 48 h under different concentrations of *Trapa natans* (0% control, 1%, 2%, and 5%) to determine time and dose dependent effects.

#### Zone of inhibition (mm) after 24 h and 48 h under various concentrations of *Trapa natans*

Table 2 summarizes the mean diameters of the zones of inhibition with *S. mutans*, *Lactobacillus* spp., and *E. coli* after 24 and 48 h. The results showed that incorporating *Trapa natans* powder in both GC Gold Label 2 and Shofu GIC improved their antimicrobial activities when measured at various concentration and time intervals.

**Table 2:** Mean diameter (± standard deviation (SD)) of zones of inhibition (mm) in disk diffusion assays against *Streptococcus mutants, Lactobacillus* spp., and *Escherichia coli* after 24 h and 48 h using GICs (GC Gold Label 2 and Shofu) modified with different concentrations of *Trapa natans.*

Treatment	GIC Brand	*Trapa natans* (%)	Time	*S. mutans* mean ± SD	*L. bacillus* mean ± SD	*E. coli* mean ± SD	Combined mean ± SD
1A	GC Gold Label 2	0%	24 h	2.10 ± 0.12	2.15 ± 0.10	2.15 ± 0.08	2.13 ± 0.10
			48 h	2.10 ± 0.95	2.20 ± 0.90	2.18 ± 0.96	2.16 ± 0.94
1B		1%	24 h	2.05 ± 0.11	2.12 ± 0.09	2.14 ± 0.10	2.10 ± 0.10
			48 h	2.20 ± 0.93	2.26 ± 0.95	2.24 ± 0.94	2.23 ± 0.94
1C		2%	24 h	2.18 ± 0.09	2.22 ± 0.11	2.20 ± 0.10	2.20 ± 0.10
			48 h	2.40 ± 0.94	2.46 ± 0.93	2.44 ± 0.95	2.43 ± 0.94
1D		5%	24 h	2.14 ± 0.10	2.17 ± 0.09	2.18 ± 0.11	2.16 ± 0.10
			48 h	2.35 ± 0.94	2.37 ± 0.93	2.36 ± 0.95	2.36 ± 0.94
2A	Shofu GIC	0%	24 h	2.18 ± 0.10	2.22 ± 0.09	2.20 ± 0.10	2.20 ± 0.10
			48 h	2.10 ± 0.93	2.16 ± 0.95	2.12 ± 0.94	2.13 ± 0.94
2B		1%	24 h	2.28 ± 0.10	2.32 ± 0.09	2.30 ± 0.10	2.30 ± 0.10
			48 h	2.48 ± 0.93	2.52 ± 0.95	2.50 ± 0.94	2.50 ± 0.94
2C		2%	24 h	2.42 ± 0.11	2.44 ± 0.09	2.42 ± 0.10	2.43 ± 0.10
			48 h	2.52 ± 0.95	2.54 ± 0.93	2.54 ± 0.95	2.53 ± 0.94
2D		5%	24 h	2.30 ± 0.09	2.34 ± 0.10	2.32 ± 0.10	2.32 ± 0.10
			48 h	2.52 ± 0.93	2.54 ± 0.94	2.54 ± 0.95	2.53 ± 0.94

Note: Combined mean ± SD calculated by averaging the diameters of the zones of inhibition for *S. mutans*, *Lactobacillus* spp., and *E. coli* under each treatment at each time point.

**Figure 1:**
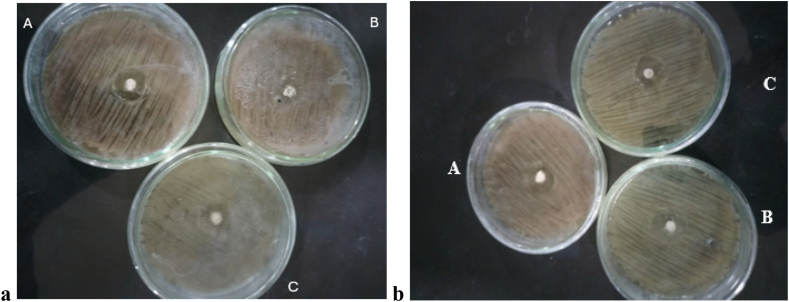
Antibacterial activities of control (0% *Trapa natans*) glass ionomer cements (GICs) against common microorganisms. (a) GC Gold Label 2 (treatment 1A) and (b) Shofu GIC (treatment 2A). Plates: A, *Streptococcus mutans*; B, *Escherichia coli*; and C, *Lactobacillus* spp.

**Figure 2:**
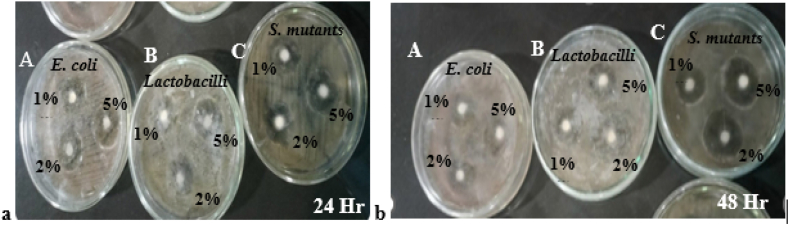
Agar disk diffusion assays using *Trapa natans*-modified GC Gold Label 2 GIC after (a) 24 h and (b) 48 h. Plate: A, *E. coli*; B, *Lactobacillus* spp.; C, *S. mutans*. Disks contained 1% (treatment 1B), 2% (treatment 1C), and 5% (treatment 1D) *Trapa natans* powder.

After 24 h, GC Gold Label 2 modified with 2% *Trapa natans* (treatment 1C) produced the largest zone of inhibition (2.20±0.10 mm), followed by the 5% modified treatment (treatment 1D) (2.16±0.10 mm). Treatment with 1% Trapa natans (1B) produced the smallest zone of inhibition at 2.10±0.10 mm, while the control (1A) treatment produced a zone of 2.13±0.10 mm (Figure 1a). Incorporating *Trapa natans* powder at various concentrations led to visible increases in the sizes of the zones of inhibition for most treatments compared to the 1% concentration (Figure 2a). The mean diameters of the zones of inhibition were comparable among the three bacterial species.

**Figure 3:**
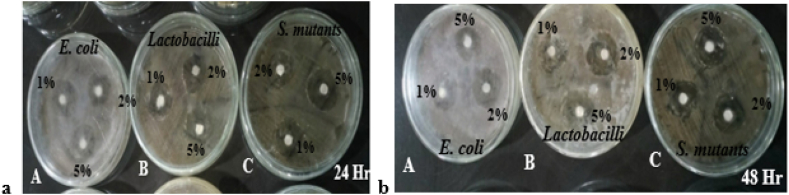
Agar disk diffusion assays using *Trapa natans*-modified Shofu GIC after (a) 24 h and (b) 48 h. Plate: A, *E. coli*; B, *Lactobacillus* spp.; C, *S. mutans*. Disks contained 1% (treatment 2B), 2% (treatment 2C), and 5% (treatment 2D) *Trapa natans* powder. Clear zones indicate the zones of inhibition measured in millimeters.

The antibacterial activities were evident under the Shofu GIC treatments after 24 h (Figure 3a). The 2% *Trapa natans* treatment (2C) produced the largest zone of inhibition at 2.43 ± 0.10 mm, followed by the 5% (2D) and 1% (2B) treatments, with mean zones of inhibition measuring 2.32 ± 0.10 mm and 2.30 ± 0.10 mm, respectively. The control treatment (2A) produced the smallest zone of inhibition at 2.20 ± 0.10 mm (Figure 1b).

The antibacterial effect was enhanced after 48 h under all experimental treatments (Figure 2b). For GC Gold Label 2, the 2% treatment (1C) produced the largest mean zone of inhibition (2.43 ± 0.94 mm), followed by the 5% (1D) and 1% (1B) treatments, with 2.36 ± 0.94 mm and 2.23 ± 0.94 mm, respectively. The control treatment (1A) produced the smallest zone of inhibition (2.16 ± 0.94 mm).

The Shofu GIC treatments had higher antibacterial effects than the GC Gold treatments after 48 h (Figure 3b). The largest zones of inhibition (2.53 ± 0.94 mm) were obtained under treatments 2C (2%) and 2D (5%), followed by 2B (1%) with 2.50 ± 0.94 mm. It should be noted that the control treatment (2A) with Shofu GIC produced the smallest zone of inhibition (2.13 ± 0.94 mm), indicating the possible positive impact of *Trapa natans* on the antibacterial activity.

Overall, these findings suggest that the antibacterial activities of both GIC brands were improved by incorporating *Trapa natans* powder, where the optimal antibacterial effects were observed at a concentration of 2% *Trapa natans*. For both GC Gold Label 2 and Shofu GIC, the minimal antibacterial activities were obtained under 0% *Trapa natans*, where the mean diameters of the zones of inhibition measured around 2.13–2.20 mm. The increases in the diameters of the zones of inhibition from 24 h to 48 h indicated that the antibacterial effects of the modified formulations were sustained and potentially enhanced over time.

#### Comparative analysis of antibacterial effects of *Trapa natan*s-modified GICs

After 24 h, post hoc Tukey tests detected no statistically significant differences in the mean diameters of the zones of inhibition between the control (0% *Trapa natans*) and any of the treatments with 1%, 2% or 5% *Trapa natans* in both the GC Gold Label 2 and Shofu GIC formulations (*p*> 0.05 for all comparisons). Thus, minimal changes in the antibacterial activities were observed under the modified experimental treatments and the changes were not statistically significant after 24 h.

However, the antibacterial activities were enhanced under *Trapa natans* incorporation after 48 h. Statistically significant differences were found in the diameters of the zones of inhibition under GC Gold Label 2 treatment with 2% *Trapa natans* (1C) and 5% *Trapa natans* (1D) compared with the control treatment (1A), where the *p*-values were 0.013 and 0.027, respectively. Similarly, Shofu GIC with 2% (2C) and 5% (2D) *Trapa natans* obtained higher antibacterial effects than the control treatment (2A), with *p*-values of 0.013 and 0.027, respectively.

These findings suggest that the antibacterial effects observed in the initial stage were not significant at 24 h, but that the impact of *Trapa natans* increased over time, especially under concentrations of 2% and 5%, indicating a time-dependent effect on the increased antimicrobial efficacy. Thus, *Trapa natans* could possibly be employed as an antibacterial additive in GIC formulations (Table 3).

**Table 3:** Post hoc (Tukey test) comparisons of mean diameters of zones of inhibition under different experimental treatments (GC Gold Label 2 and Shofu GIC) after 24 h and 48 h.

Comparison	GIC brand	Time	Mean difference (mm)	Standard error	*p*-value	95% Confidence interval (lower – upper)
**1A vs 1B**	**GC Gold 2**	**24 h**	0.03	0.107	0.989	−0.34 to 0.27
**1A vs 1C**			−0.15	0.107	0.518	−0.46 to 0.16
**1A vs 1D**			−0.09	0.103	0.818	−0.38 to 0.20
**1A vs 1B**		**48 h**	−0.22	0.094	0.14	−0.49 to 0.05
**1A vs 1C**			−0.33	0.094	0.013^a^	−0.60 to −0.06
**1A vs 1D**			−0.3	0.094	0.027^a^	−0.57 to −0.03
**2A vs 2B**	**Shofu GIC**	**24 h**	−0.1	0.107	0.989	−0.34 to 0.27
**2A vs 2C**			−0.23	0.107	0.518	−0.46 to 0.16
**2A vs 2D**			−0.12	0.103	0.818	−0.38 to 0.20
**2A vs 2B**		**48 h**	−0.22	0.094	0.14	−0.49 to 0.05
**2A vs 2C**			−0.33	0.094	0.013^a^	−0.60 to −0.06
**2A vs 2D**			−0.3	0.094	0.027^a^	−0.57 to −0.03

a *p*-value <0.05 denotes a significant difference.

## Discussion

The results obtained in the present study highlight the enhanced antibacterial potential of GIC when modified with *Trapa natans* extract. The findings confirmed dose- and time-dependent improvements in the antimicrobial activities against key oral pathogens comprising *S. mutans*, *Lactobacillus* spp., and *E. coli*. These microorganisms are well-established contributors to dental caries, plaque formation, and secondary oral infections, making them ideal targets for evaluating the antimicrobial potential of restorative materials.

The null hypothesis considered in the present study was that incorporating *Trapa natans* powder into GC Gold Label 2 and Shofu GICs would not alter their antibacterial activities. However, the results indicated that the antimicrobial activities were significantly enhanced, particularly under higher concentrations and extended incubation times, and thus the null hypothesis was rejected. The selected concentrations were based on preliminary pilot testing and previous studies of herbal-modified GICs, which indicated that low concentrations may be insufficient for antimicrobial effects, whereas higher concentrations could potentially affect handling, consistency, or the powder:liquid ratios recommended by manufacturers.^15^^,^^16^ The findings obtained in the present study showed that concentrations of 2% and 5% resulted in the optimal enhancement of antibacterial effects without influencing the material's usability.

Agar disk diffusion assays showed that using both the GC Gold Label 2 and Shofu GIC formulations, the diameters of the zones of inhibition increased after incorporating *Trapa natans*, particularly concentrations of 2% and 5%. It should also be noted that the enhancement of the antibacterial activity was more pronounced after 48 h compared with 24 h, indicating the sustained and progressive release of the active antimicrobial constituents. Similarly, previous studies also suggested that the efficacy of plant-based antibacterial agents often improved after prolonged exposure.^15^

The enhanced antibacterial activity could probably be attributed to the high phenol, flavonoid, and coumarin contents of *Trapa natans*. Phenolic compounds disrupt bacterial cell membranes and denature proteins, and flavonoids interfere with DNA synthesis and metabolic pathways.^17^^,^^18^ The presence of these two mechanisms distinguishes *Trapa natans* from other herbal GIC modifiers, such as chitosan (primarily electrostatic interactions) or neem (oil release), which rely on different antimicrobial mechanisms. Integrating *Trapa natans* into the GIC matrix retained the acid–base setting reaction, as well as allowing the gradual release of phytochemicals, maintaining both the material's integrity and antibacterial efficacy.

Among the experimental treatment formulations tested in the present study, the antimicrobial performance was slightly superior for Shofu GIC, suggesting a possible synergistic effect between the GIC composition and phytochemical profile of *Trapa natans*, which could be attributed to the higher affinity of *Trapa natans* compounds for integrating within the matrix of Shofu GIC, or possibly more favorable release kinetics. These findings support previous studies that reported enhanced antibacterial activities against cariogenic bacteria using GICs modified with herbal additives such as *Olea europaea*, *Ficus carica*, and *Salvadora persica.*^16^

The presence of these compounds likely explains the observed improvements in microbial inhibitory activity, particularly against *S. mutans*, which is the primary etiological agent in dental caries.^18^

Statistical analysis (post hoc Tukey) revealed significant differences between control and experimental groups at 48 h, specifically for the 2% and 5% *Trapa natans* concentrations. Although no significant differences were observed at 24 h, the trend toward improved efficacy suggests a time-dependent release of active components. This delayed enhancement is a favorable characteristic for restorative materials requiring long-term antimicrobial action.

The use of *Trapa natans* as a natural additive is particularly interesting given increasing concerns regarding antimicrobial resistance and the need to use biocompatible, non-toxic materials in dentistry. Compared with synthetic agents, such as chlorhexidine or antibiotics, plant-based extracts are safer and more sustainable alternatives with potentially fewer adverse effects.

### Strengths

This study is among the first to evaluate *Trapa natans* as a modifier for use in conventional GICs. The present study tested two commercially available GIC brands and assessed the incorporation of multiple concentrations of *Trapa natans* to determine dose-dependent effects. Antibacterial tests targeted clinically relevant cariogenic microorganisms.

### Limitations

This study was conducted under in vitro conditions that did not fully simulate the oral environment. Mechanical properties, long-term fluoride release, cytotoxicity, and biofilm inhibition were not evaluated. Only three bacterial strains were tested.

Further in vivo studies and comprehensive material property assessments are recommended to confirm the clinical applicability of *Trapa natans*.

## Conclusion

This study tested a novel approach by incorporating *Trapa natans* (water chestnut) powder into conventional GICs (GC Gold Label 2 and Shofu) to enhance their antimicrobial properties. The results demonstrated that modification of the GICs with *Trapa natans* obtained concentration-dependent increases in the antimicrobial activities against *S. mutans*, *Lactobacillus* spp., and *E. coli*, particularly at *Trapa natans* concentrations of 2% and 5%. It was notable that the antibacterial efficacy of Shofu GIC was comparatively greater than that of GC Gold Label 2 at all concentrations and time points. These findings suggest that *Trapa natans* has potential for use as a natural, plant-based additive to improve the antimicrobial properties of restorative dental materials. Future studies of *Trapa natans*-modified GICs should involve evaluating their long-term antibacterial activities and biofilm inhibition in vivo, assessing their mechanical performance and durability, conducting comprehensive biocompatibility and cytotoxicity assessments, and determining the optimal concentration ranges for maximum antibacterial activity without affecting the handling or material properties.

## Ethical approval

Approval was obtained from the Institutional Ethics Review Committee (BDC/ERC/2023/052; 15 June 2023).

## Authors contributions

Dr. AI contributed to the study design, methodology, sampling, data collection, and data processing. Prof. Dr. SK supervised the study, while Dr. ZA served as co-supervisor. Dr. RF performed statistical analysis, interpreted the results, and drafted the manuscript. Dr. MKHU and Dr. AA critically reviewed the manuscript and contributed to its improvement. All authors have critically reviewed and approved the final draft and are responsible for the content and similarity index of the manuscript.

## Availability of data and materials

Available from corresponding author on reasonable request.

## Source of funding

This research did not receive any specific grants from funding agencies in the public, commercial, or not-for-profit sectors.

## Conflict of interest

The authors have no conflict of interest to declare.
